# Supplementary material for: Barriers and facilitators to the delivery of age-friendly health services in Primary Health Care centres in southwest, Nigeria: A qualitative study
Source: PLoS One. 2024 Mar 19;19(3):e0288574. doi: 10.1371/journal.pone.0288574 (PMC10950227; doi:10.1371/journal.pone.0288574)
Supplement: S1 File — (DOCX) [file pone.0288574.s001.docx]

**INTERVIEW GUIDE AND PROMPTS**

**Pre-discussion briefing**

Welcome & Introduction

Thank you for agreeing to speak with me today, I appreciate you taking the time out of your day to participate in this study. I am working with the research team from the College of Medicine, the University of Lagos and the Northwestern University to better understand the care of older people in PHCs in Lagos State.

- *Action*: Please provide the information sheet and consent form to be read and signed

Signed consent form

Permission to voice record

To allow me to focus on what you’re saying instead of taking notes, I would like to use a voice recorder that will allow me to transcribe our meeting at a later time. At no time during my reporting or when I summarise what is discussed will you be identified, except by discipline where appropriate. Do you agree to be voice recorded?

Participant consented to voice recording  Participant did not consent to a voice recording

- *Action*: If the participant is in agreement, commence voice recording. If the participant declines, a voice recording will not be undertaken.

**[Do not read aloud – for internal reminder of the purpose of the interview]**

**Interview will inform Aim 3:**

To explore the barriers and facilitators to the delivery of AFHSs in PHCs in Lagos State, Nigeria.

Topic introduction

We recently conducted a survey in selected comprehensive PHCs in Lagos State to assess the age-friendliness of its services, staff and the environment for older adults. We’d like to understand your thoughts on this subject.

1. According to the WHO, healthy ageing is the process of developing and maintaining the functional ability that enables wellbeing in older age.
2. What do you know about healthy ageing?
3. At the level of the PHCs
4. What policies are you aware of that are relevant to support the delivery of healthy ageing services to older people in our PHCs?
   1. Which is/are helpful in the delivery of services to older people?
   2. Which is/are helpful in supporting the uptake of services by older people?
   3. Which are not helpful for the above?
5. How important do you think it is to strengthen care for older people (beyond managing Non-Communicable Diseases) in our PHCs?
   1. Probe: If important, why is it important? Give specific example(s)
   2. If not important, why not? What is/are higher priority(ies) in PHCs?

What are some successful examples of PHCs delivering health services in ways that are responsive to older people?

- 1. Can you give specific examples
  2. What are the challenges in delivering these health services?

1. In your opinion, what are the interventions needed to better meet the service and support needs that are unique for older adults in the PHCs?
2. Probe for details of each intervention mentioned
3. What about older adults with cognitive decline, frailty
4. What do you think about the current financing for service delivery for the care of older persons in our PHCs for example staffing, and supplies?
   1. Probe-is it enough or not enough?
   2. Where is more financing needed? Why?
   3. What are sources of financing?
   4. What are challenges with financing? How can these be overcome?

-

1. How well are data from the PHC being used in understanding the care of older people?
   1. What are examples of the use of data?
   2. What are reasons for non-use of data? Probe: not able to disaggregate? Not a priority? What else?
   3. How does this differ from the way it is used for other age groups?
2. What challenges do you think older people have regarding physical access at the centres?
3. Probe: For example, the WHO recommendation for age-friendly PHCs gives a minimum width of 900mm for all doors and corridors to allow for wheelchair users? Is this working or not? Why?
4. From our assessment, some PHCs did not have ramps at their entrances and where available, some did not meet the required dimensions- how much of a challenge is this? How hard would this be to address?
5. What is the need for community-based services for older people in Nigerian communities?
   1. Probe: What are the areas of need?
   2. Are they being met?
   3. If yes, can you give some examples
   4. If not, what are the challenges?
6. What types of community education and demand generation is needed for increasing the number of older people using the PHCs?

**Post-Discussion Debrief**

Summary of discussion

Thank you all for the insightful discussion. It seems to me like the main points were that… [Summarise discussion] Did I miss anything?

Is there anyone else I should be talking to? If Yes, who?

Thank you for participating.

- *Action*: End voice recording.
