# Supplementary material for: Barriers and facilitators to the delivery of age-friendly health services in Primary Health Care centres in southwest, Nigeria: A qualitative study
Source: PLoS One. 2024 Mar 19;19(3):e0288574. doi: 10.1371/journal.pone.0288574 (PMC10950227; doi:10.1371/journal.pone.0288574)
Supplement: S2 File — (DOCX) [file pone.0288574.s002.docx]

**S2 Table: Coding Tree**

| Parent Code | Child Code | Description |
| --- | --- | --- |
| Access to PHCs |  | Mention of getting to the PHC. Examples might include the location of PHCs, geographic coverage, bus schedules, etc. Do not talk about financial access or physical environment of the building inside (those have separate codes). |
| Advocacy |  | Meeting with individuals with monetary or political agencies to influence them to create, fund and implement policies and services for older adults |
| Affordability |  | Of care and services for patients. Patient's affordability. |
|  | Financial Aid | Money given to individuals to cover the costs of care. Welfare packages. |
|  | Free services | Free services or programs for older adults either in the PHC building or in other community spaces |
|  | Health Insurance | Discussion of individual uptake and use of insurance and discussions of PHC/provider acceptance of insurance. |
| Availability |  | Availability of appropriate, tailored services and care for older adults to meet their specific needs. |
|  | PHC programs and services | appropriate, tailored older adults programs and services in the PHC facility |
| Barrier |  | Apply as a secondary code to all discussions of things that are barriers. |
| Budget |  | Budget line item to fund older adult services and programs. Can also include talk about the cost of things to the health system or PHC (not cost to the patient) '-' things like hiring staff, infrastructure changes, training, etc. |
| Community Delivery |  | Providing services in the community to address healthcare needs (e.g., healthcare workers visiting at home) |
|  | Social needs | Social support services in the community (e.g., support with housing, feeding, etc.) |
| Community Education and Mobilization |  | To create awareness on health care services for older adults. |
| Community programs and services |  | Appropriate, tailored older adults programs and services in the community (outside of the PHC facility) |
| Data Availability |  | Ability to access/obtain data |
| Data Use |  | Ability to use data to guide care and funding policies and practices for older adults. Also discussion or examples of use in this way. |
| Facilitator |  | Apply as a secondary code to all discussions of things that are facilitators. |
| Guidelines |  | Discussion of the needs for or current guidelines for appropriate care/services for older adults |
| Healthcare Needs |  | Discussion of older adults healthcare needs |
|  | Curative/  Treatment | Discussion of curative/treatment needs |
|  | Health Promotion/ Prevention | Discussion of Health promotion and prevention programming needs |
|  | Other Programming  Interventions | Special and dedicated services for the elderly not covered under one of the other buckets of needs |
|  | Patient Education | Enlightening/Preparing people about the expectations that come with ageing. |
|  | Rehabilitative | Discussion of rehabilitative needs |
| Healthy Aging Definition |  | Discussion of what healthy aging means to the person. |
| Needs Assessment |  | Understanding the needs and gaps of care in this population (e.g., surveys, review of data) |
| Past success |  | Description of programs or interventions that worked in the past. Or discussions of ways older adults needs were prioritized and solutions implemented. |
| Physical Accessibility |  | Discussion of the built environment of the building (e.g., ramps, railings, hallway/door width etc.) and/or making changes to bring the physical space into alignment with WHO standards. |
| Policies |  | Mention of the types of policies needed or present to address older adults needs and support older adults focused care and services |
| Provider Training and Education |  | Training and education needed or provided to PHC providers to ensure appropriate care and programming for older adults |
| Skilled personnel |  | The presence or absence of an adequate workforce that has gone through training on how to specifically care for the older persons. |
| Socialization |  | An avenue for older people to go out of their homes and interact with others |
| Stakeholder Engagement |  | Any work to engage key stakeholders with the goal of getting input and ideas into the plans/design, and obtain buy'-'in from all stakeholders about the importance of older adults programming and services (including older adults, community members, PHC staff and providers, funders, government officials, etc.). This code will also cover discussions of strategies such as community outreach to inform older adults (and other stakeholders) about services to increase their use. |
