# Supplementary material for: Barriers and facilitators to the delivery of age-friendly health services in Primary Health Care centres in southwest, Nigeria: A qualitative study
Source: PLoS One. 2024 Mar 19;19(3):e0288574. doi: 10.1371/journal.pone.0288574 (PMC10950227; doi:10.1371/journal.pone.0288574)
Supplement: S3 File — (DOCX) [file pone.0288574.s003.docx]

**S3 Additional interview themes: Barriers and facilitators to the delivery of age-friendly services**

| **Theme** | **Subtheme** | **Quotes** |
| --- | --- | --- |
| Staff limitations | Staff shortage | *“If we have many helping hands to assist, human resource is a challenge, so, if we have many doctors, we still need many doctors at the PHC, you know, the ratio of doctors to clients is still very low in this part of the world, we are even trying in Lagos State when compared to other States but, it is still not enough. So, for us to break that challenge, that means we need more hands, so, if not they will still come and queue, may be there for long time before being attended to.”* – P4 |
| Insufficient programing to meet needs | Home visits for check-ups | *“The areas of need is like what I said the other time that defaulter tracking, that they should be checking on them, home visit, that they should be visiting them at home checking on them like weekly bases you know elderly some of them live alone like in all these our estates they live alone, they don't have anybody living with them again, so it is very very important for the elderly in the community to be attended to, at least it will help them”-* P13 |
|  | Tailored care | *“whatever intervention you want to carry out, it has to be community based and the truth of the matter is in carrying out such community based Interventions, it must be centered around each individual because all of them have different needs and at the same time it must also weave together all those individuals within the community, so, it is not just going to be about having community based intervention, you know that will just be focused only on the elderly, it must be focused on the elderly, it must also be done in such a way that it will help the elderly to integrate with the community that they have found themselves in, because that is the only thing that will make it sustainable in the long run.”* – P8 |
| Physical building isn't serving older adults well | Buildings are not purpose built | *“I will say may be in the last ten years, the way the PHCs have been structured has been irregular, there are times when in a local government the chairman will just wake up in a day and decide to donate a building to the hospital or even build a PHC facility without the input of any health worker or any agency anywhere, it may just be between him and may be one or two officers within the local government and at the end of the day the health worker is faced with a building that's not built with a hospital setting in mind.”-* P2 |
|  |  | *“Yes most of the places do not have ramp” -* P13 |
| Data not disaggregated for older adults |  | *“Like we said we disaggregate data very well, we do a lot of disaggregation according to like I said priorities, women of child bearing age, for maternal mortality, children, you know for child mortality so we do all those, but unfortunately like I said prior, the primary health care level is mainly about communicable diseases and curbing those, and we treatments of minor ailments so if we want to talk about data, and the focus of that, it’s best to ask at the ministry of health, they are the ones to collate and collect those data and disaggregate appropriately”* - P11 |
|  |  | *“Well, the answer will be that we really don’t, honestly speaking, we really don’t aggregate data for the elderly.”* - P8  *“…there is something that we call the Monthly Summary in the National Health Management Information System, the source data is the Facility Register and the age group there is 0 to 5years, 5 to18years then 18years and above…..So, you cannot analyze beyond what your system allows you to do”.* - P1 |
| Financial limitations |  | *“You know some by the time they see the doctor they ask them to do test they may say they don’t have funds like the one I referred from my church yesterday to a PHC, he was very weak and there was no doctor there to attend to him, when they called me, I said they should refer him to the flagship at another place when he got to there, they wanted to attend to him but due to lack of fund they had to refer him to a General Hospital, I have been looking for him since yesterday now, I called my pastor that night that this man I want you to help me check on him, he sent somebody to his house they said he left his home in the morning that he is going to the hospital and since then he did not come back but to God be the glory the pastor called me this afternoon that he saw him right on the street, moving out of the street that they had discharged him. If for instance we had funds and there is a finance that is attached to elderly like that they will attend to him without any problem” -* P13 |
| **Facilitators to the delivery of age-friendly services** | | |
| **Theme** | **Subtheme** | **Quotes** |
| PHC Mission |  | *I wouldn’t say we have special clinic, you know for the adults but what we have is, we have a PHC Structure that cares for all age groups, starting from newborn up to old age.* - P8  *We have a PHC Structure that cares for all age groups, starting from newborn up to old age.* - P8 |
| Opportunities for the enhancement of older adult care |  | *“Lagos State makes it easy for them to be able to access free drugs, we need to revitalize that, we need to bring it on board, so that they can come to the facility easily and get care even when they don’t have anybody, and that is the reason why the 1% health insurance mapping should be equally extended to them so that they can come to the facility whether they have money or not, they should be enumerated and enrolled for this care of service” -* P10 |
